# Gall Bladder Perforation During Percutaneous Nephrolithotripsy: Lessons Learned

**DOI:** 10.7759/cureus.69909

**Published:** 2024-09-22

**Authors:** Nakul Aher, Subash Kaushik TG, Rubina Singh, Hariharasudhan Sekar, Sriram Krishnamoorthy

**Affiliations:** 1 Urology, Sri Ramachandra Institute of Higher Education and Research, Chennai, IND

**Keywords:** biliary peritonitis, complications, gall bladder injury, laparoscopic cholecystectomy, percutaneous nephrolithotomy (pcnl)

## Abstract

Percutaneous nephrolithotripsy (PCNL) is the recommended procedure for patients with large and complex renal calculi. Visceral injuries are rare complications of this procedure. A distended gall bladder (GB) that abuts the right kidney is more prone to needle puncture-related injuries. A GB injury leading to peritonitis portends a poorer prognosis. We report a case of GB perforation that happened after an otherwise uneventful PCNL, which was managed with a prompt intraoperative decision of performing laparoscopic cholecystectomy. The initial puncture yielded a straw-colored, gelatinous aspirate, alerting the treating surgeon. After the completion of the PCNL, a diagnostic laparoscopy was performed. The GB was found to be injured and a bile leak was observed. Elective cholecystectomy was performed. Awareness of this potentially lethal complication and a high index of clinical suspicion is mandatory, as early diagnosis and prompt management can prevent mortality in such patients.

## Introduction

Percutaneous nephrolithotomy (PCNL) is a universally accepted minimally invasive surgical procedure for the management of large renal stones. Nearly one-third of the patients in any actively functioning urology center have stone-related problems [1]. The standardization of the grading of PCNL-related complications by the Clavien-Dindo grading system has greatly facilitated auditing and classification of the severity of complications of PCNL [2]. The incidence of major complications may be higher in teaching hospitals, as the trainees and junior consultants may do more of these procedures.

Life-threatening complications like hematuria, pleural, splenic, colonic, duodenal, and ileal injuries have been documented but are rare [3-5]. Right-sided PCNL must be performed with greater caution given its proximity to the inferior vena cava, gall bladder (GB), and the second part of the duodenum. GB perforation is one of the rare visceral complications of PCNL carrying high morbidity and mortality [6]. Caution should be exercised if a straw-colored or gelatinous aspirate is noticed at the time of the initial puncture. In cases like these, timely identification and treatment are vital because biliary peritonitis can become fatal if treatment is delayed [7,8]. We report a rare case of GB perforation during PCNL. We discuss the methods to anticipate this lethal complication, the measures to be taken if there is a strong suspicion of such complication, and the lessons learned from this incident.

## Case presentation

A 68-year-old diabetic female was admitted for low-grade fever and burning micturition for one month. Her body mass index (BMI) was 21.6 (weight=61 kg, height=1.68 meters). Her hemoglobin (Hb%) was 7.3 mmol/L, total leucocyte count was 5.4x10 9 cells per liter, serum creatinine was 50 µmol/L, and serum electrolyte and coagulation profile on admission were normal. Urine microscopy showed plenty of pus cells. The urine culture was sterile.

Investigations

**Figure 1:**
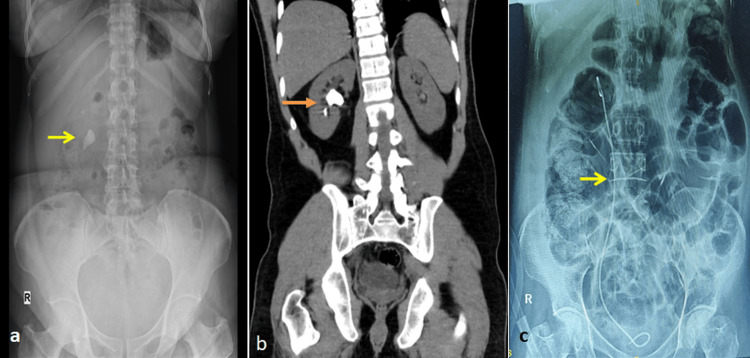
Right renal calculus that needed PCNL a) Plain KUB X-ray showing a 3 cm-sized right renal calculus (yellow arrow). b) Computed tomography (CT) of the abdomen, confirming the presence of hard-right renal pelvic calculi of size 3.5 cm with 1035 HU (orange arrow). c) illustrates the post-operative plain X-ray abdomen-erect view, showing the DJ stent in position (yellow arrow) and dilated bowel loops, consistent with paralytic ileus. PCNL: percutaneous nephrolithotripsy; KUB: kidney, ureter, and bladder; DJ: double-J

A kidney, ureter, and bladder (KUB) X-ray showed a 3 cm-sized right renal calculus, and computed tomography (CT) of the abdomen confirmed the presence of hard-right renal pelvic calculi of size 3.5 cm with 1035 HU (Figure 1).

Treatment

**Figure 2:**
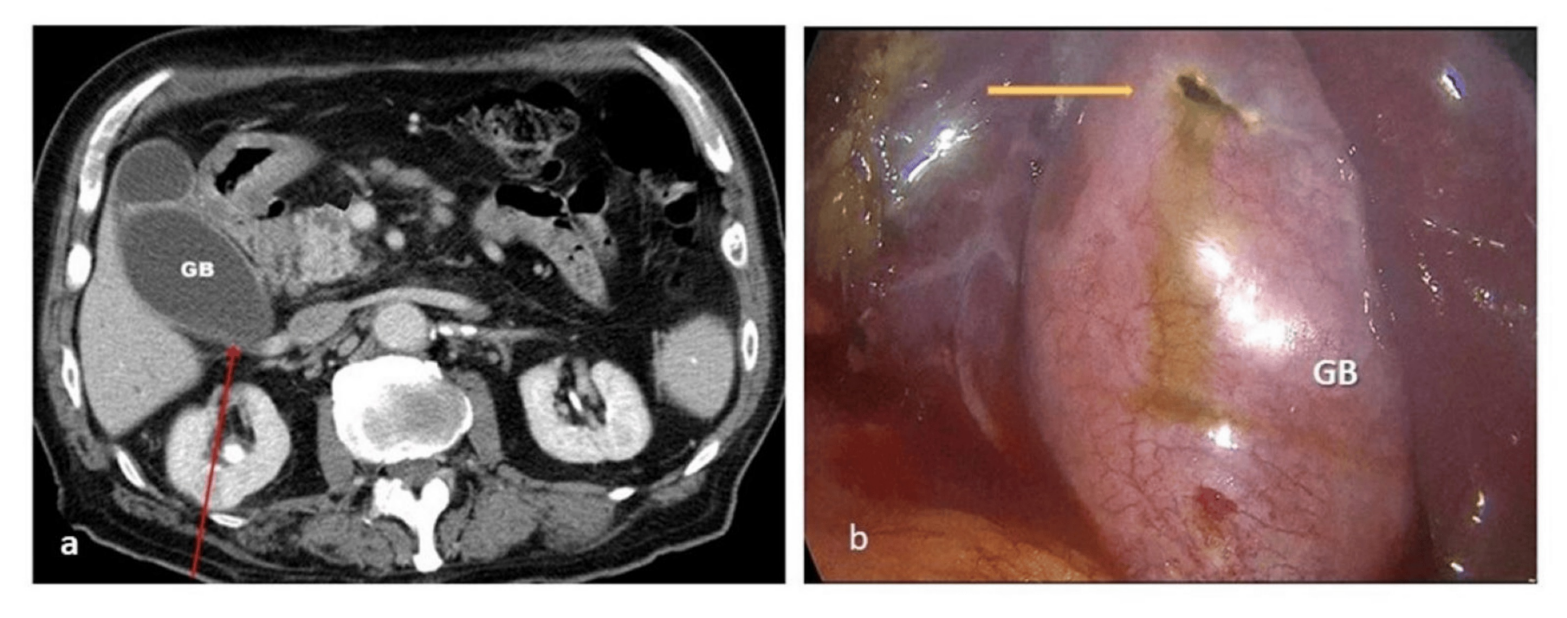
Relationship between the right kidney and the gall bladder (GB), and an intra-operative finding of the diagnostic laparoscopy a) Axial computed tomography (CT) image of the abdomen showing the relationship between the right kidney and the GB (red arrow). b) Intra-operative finding of the GB perforation site with bile leak (yellow arrow).

Under general anesthesia, in a prone position, by bull's eye technique, a lower calyceal puncture was made using a 16G, 15 centimeters long two-part trocar tip initial puncture needle. A straw-colored, gelatinous fluid was aspirated initially. The needle was immediately readjusted and the guidewire was secured within the collecting system. PCNL was done with complete stone clearance. The overall procedure time was 30 minutes. Following the PCNL, a diagnostic laparoscopy was carried out since the initial puncture revealed a straw-colored aspirate. The duodenum was normal. There was a bile leak from the surface of the GB. Figure 2b shows an intra-operative finding of the GB perforation site. A surgical gastroenterologist's opinion was sought, elective cholecystectomy was performed, and peritoneal lavage was done.

Outcome

The percutaneous nephrostomy (PCN) tube was removed on the first postoperative day. Figure 1c illustrates the post-operative plain X-ray abdomen-erect view, showing the double-J (DJ) stent in position and dilated bowel loops, consistent with paralytic ileus. The patient recovered well and was discharged on the third postoperative day.

Lessons learned

The authors had a similar experience a few years back, where there was a delay in the diagnosis of bile leak and biliary peritonitis. The authors emphasize that it is important to keep in mind this potentially lethal but preventable complication while doing right-sided PCNL. The GB that is over-distended as a result of overnight starvation may occasionally come in the way of the line of needle puncture, especially whenever one overshoots the target. In such cases, it is prudent to either stop the procedure and perform cholecystectomy or complete the PCNL procedure and do cholecystectomy immediately thereafter. Prompt action can be lifesaving. 

## Discussion

PCNL is a standardized, safe, and efficient method used to treat major kidney stones. Biliary peritonitis secondary to a GB injury is a lethal but potentially correctable complication in patients undergoing PCNL. An over-distended GB due to overnight starvation, especially in those with lower BMI, is at a higher risk, as the GB closely abuts the hilum of the right kidney. Right-sided percutaneous renal access increases the risk of gallbladder injuries during the initial puncture [9,10].

**Figure 3:**
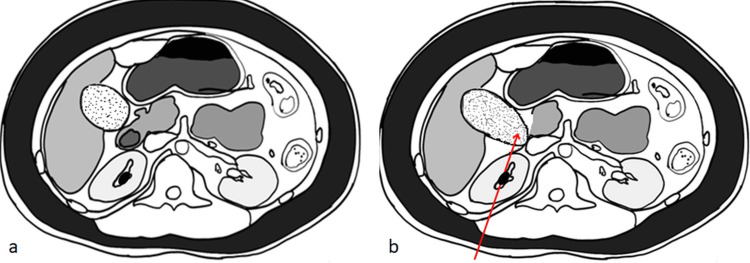
Relationship between the gall bladder (GB) and the right kidney a) (Non-distended GB) and b) (distended GB) provide a schematic representation of how an over-distended GB might get injured during the initial puncture. Image Credit: Authors of this study

Figure 3 gives a schematic diagram of the relationship between an over-distended GB and the right kidney. This illustration depicts how a right-sided PCNL can cause injury to the GB when it is over-distended.

Unlike colonic injury, where spontaneous seal-off may happen, GB injury may not seal off by itself. A continuous biliary leakage will cause biliary peritonitis, which presents initially as reflex paralytic ileus, new-onset dyspepsia, vomiting, and vague non-specific abdominal pain, often delaying a definitive diagnosis [11]. A diagnosis is more often made after biliary peritonitis sets in [12 ]. The presentation in most cases is delayed, as the symptoms worsen when the patient starts taking solid food, and the GB contracts.

When the diagnosis is made early, the proposed procedure can be deferred and minimally invasive techniques like endoscopic retrograde cholangiopancreatography (ERCP) and stent placement attempted to minimize morbidity [13]. If the diagnosis is made late, exploratory laparotomy with or without cholecystectomy gives good results [14]. The reported incidence of morbidity and mortality due to post-operative biliary peritonitis varies from 2 to 15% and 1 to 6%, respectively [15].

In a thorough PubMed literature search, only 12 published cases of GB perforation have been reported till now. Table 1 summarizes the list of all the previously published literature on GB injury during PCNL.

**Table 1:** Summary of previously published reports of gall bladder injuries during PCNL ERCP: endoscopic retrograde cholangiopancreatography; CBD: common bile duct; PCNL: percutaneous nephrolithotripsy

Clinical study	No. of patients	Clinical setting	Intervention done	Outcome	Timing to diagnosis
Ricciardi et al. [8]	1	a) Hemorrhagic shock. b) Septic shock with high output biliary fistula	a) Exploratory laparotomy and right nephrectomy. b) External biliary derivation, duodenal exclusion, and gastro-jejunal anastomosis	Successful	a) 24 hours. b) After seven days
Patel and Nakada [9]	2	1) Pain abdomen with sepsis. 2) Pain abdomen	1) Exploratory laparotomy and cholecystectomy, nephrostomy. 2) Laparoscopy and cholecystectomy, nephrostomy	1) Successful. 2) Successful	1) 48 hours. 2) 48 hours
Yadav et al. [10]	1	Pain abdomen	ERCP + CBD stenting	Successful	24 hours
Ranjan et al. [12]	1	Abdominal distention with hypotension	Exploratory laparotomy + cholecystectomy	Unsuccessful	48 hours
Martin et al. [16]	1	Pain abdomen and distention, and fecaloid vomiting	Exploratory laparotomy and cholecystectomy	Successful	48 hours
Saxby et al. [17]	1	Pain abdomen	Exploratory laparotomy and cholecystectomy + insertion of a T-tube	Successful	48 hours
Kontothanassis et al. [14]	2	1) Pain abdomen. 2) Pain abdomen and distension	1) Exploratory laparotomy and cholecystectomy + insertion of a T-tube. 2) Exploratory laparotomy and cholecystectomy	1) Successful. 2) Successful	1) 48 hours. 2) 12 hours
Sharma et al. [18]	1	Pain abdomen	Percutaneous drainage	Successful	Intraoperatively
Rahnemai-Azar et al. [19]	1	Pain abdomen with sepsis	Percutaneous cholecystostomy	Successful	Intraoperatively
Patil et al. [20]	1	Abdominal distension	Conservative management	Successful	Intraoperatively
Our study	1	Abdominal distension	Laparoscopy and cholecystectomy, nephrostomy	Successful	Intraoperatively

In our instance, a timely intraoperative choice to perform laparoscopic cholecystectomy along with the otherwise uneventful PCNL treated the GB perforation, averting a potentially deadly complication of biliary peritonitis. A lack of knowledge/awareness of this potentially lethal entity can be very costly in the end. The clinical picture may get worse if treatment is postponed. The development of ileus and sepsis due to biliary peritonitis comes under Grade V in the modified Clavien grading system, prompting earlier intervention.

As biliary peritonitis is an acute surgical emergency, the treating urologists and surgeons need to adopt earlier intervention in cases of inadvertent GB injury during PCNL.

## Conclusions

PCNL is a common procedure performed by urologists. Caution should be exercised while performing right-sided PCNL, given its proximity to the inferior vena cava, GB, and duodenum. A gelatinous or bilious aspirate during initial puncture should forewarn a possible GB injury. It is prudent to defer PCNL and perform diagnostic laparoscopy with or without cholecystectomy if a GB puncture is suspected. A GB injury manifests after 24-48 hours of surgery, especially after the patient is started on solid oral feeds. A delayed presentation portends a poorer prognosis. Because biliary peritonitis can be fatal, saving a patient's life requires early detection, appropriate diagnosis, and fast treatment.
